# Defining screening criteria and ranking in-row and inter-row cover crops for irrigated vineyards using a hybrid AHP–TOPSIS model

**DOI:** 10.3389/fpls.2025.1695610

**Published:** 2026-01-26

**Authors:** Mehdi Sharifi, Abbas Sayyad, Eman El Sayed

**Affiliations:** 1 Summerland Research and Development Centre, Agriculture and Agri-Food Canada, Summerland, BC, Canada; 2 Lambton College of Applied Arts and Technology, Applied Research and Innovation Program, Sarnia, ON, Canada; 3 School of Engineering, University of Guelph, Guelph, ON, Canada

**Keywords:** *Trifolium repens*, *Lens culinaris*, *Lolium perenne*, multicriteria decision analysis, *Raphanus sativus*, wine grape

## Abstract

Cover crops are increasingly important in sustainable vineyard management, yet species selection remains site-specific and challenging. We evaluated candidates in two commercial organic vineyards in the semi-arid Okanagan Valley, British Columbia—CFF (13-year Merlot) and KOW (10-year Zweigelt)—using a hybrid multicriteria decision analysis (MCDA) [Analytic Hierarchy Process–Technique for Order Preference by Similarity to Ideal Solution (AHP–TOPSIS)]. Nine in-row and 15 inter-row treatments (annuals and perennials) were evaluated in the 2019 growing season. Field measures included biomass, ground cover, interference with the fruiting zone, invasiveness, pest/disease effects, drought and winter tolerance, and traffic tolerance. In both vineyards, risk of invasiveness (~43–60%) and interference with fruiting zones (~30%) were the most influential criteria. The top under-vine annuals were *Lens culinaris* (spring lentil), *Brassica napus* cv. Winfred, and *Brassica rapa* (purple-top turnip) with high relative closeness (RC_i_ = 0.87–0.96). Among perennials, Ladino *Trifolium repens* cv. Crescendo ranked the highest (RC_i_ ~ 0.84), supporting its use as a durable under-vine cover. For inter-rows, *Pisum sativum* + *Secale cereale* (pea–rye) led at CFF (RC_i_ = 0.89) and placed second at KOW, *Trifolium incarnatum* ranked second at CFF (RC_i_ = 0.83), and *Trifolium alexandrinum* led at KOW (RC_i_ = 0.94). Other annual clovers were intermediate (RC_i_ = 0.79–0.88), performing best on finer-textured, cooler, moister sites. The leading perennial inter-row mix was *Lolium perenne* + *Raphanus sativus* var. *longipinnatus* (tillage radish; RC_i_ = 0.94), followed by a *Festuca* spp. mix (RC_i_ = 0.65–0.69). This MCDA delivers clear, literature-aligned rankings and a data-driven framework to guide regionally adapted cover-crop choices and future web-based decision tools.

## 1 Introduction

Cover crops are increasingly recognized as essential for sustainable viticulture. Vineyards worldwide face intertwined challenges such as herbicide-dependent weed control with rising resistance, soil compaction and slope erosion, nutrient losses, and declines in soil organic matter and biodiversity (Abdalla et al., 2019; Abad et al., 2021a; Liebhard et al., 2024). Evidence shows that well-chosen cover crops suppress weeds, improve soil structure and infiltration, reduce erosion and nutrient leaching, and enhance beneficial habitat and biodiversity (Abad et al., 2021a; Hasanaliyeva et al., 2024; Liebhard et al., 2024; Lines et al., 2024). Despite growing interest, irrigated vineyards still lack field-validated, site-specific criteria for the selection of cover crop species. The use of unsuitable species can attenuate expected ecosystem services and introduce unintended disservices. Vineyard-specific, field-validated multicriteria ranking frameworks are scarce; most recent studies have tested species in isolation instead of developing integrated decision-support models (Abad et al., 2023; Sharifi et al., 2024). Data-driven, multicriteria decision analysis can explicitly encode trade-offs and produce transparent, reproducible rankings for cover crop species (Sathiyamurthi et al., 2024). Embedding such models in web-based decision-support tools could hasten adoption and enable region-specific tailoring, complementing existing platforms such as Wine Australia’s Cover Crop Finder (Wine Australia, 2025).

Cover crop benefits depend on selecting species adapted to site conditions and placement. Vineyard floor management includes in-row (under-vine) and inter-row (alley) distinct zones, which differ ecologically and operationally. Inter-row covers are widely used to reduce erosion and enhance soil fertility while avoiding direct competition for light or trellis space (García-Díaz et al., 2018). Under-vine covers can compete for water and nutrients and impede operations, yet they are gaining traction as herbicide/tillage alternatives for weed control and for vine vigor regulation (Vanden Heuvel et al., 2021). Reported effects include modest vine growth reductions (e.g., ~8% yield in some cases) without consistent fruit-quality penalties, alongside added services such as soil protection and biodiversity (Abad et al., 2021a, b). Consequently, in-row species must be low-growing and tightly managed, while inter-row species can be taller/robust because they are readily mowed and confined to inter-rows. Life cycle strongly shapes cover crops’ performance. Annuals establish rapidly, deliver quick ground cover and weed suppression, and, when timed to grow outside critical vine demand (e.g., cool-season annuals terminated before summer), can limit competition (Jordan et al., 2016; DeVincentis et al., 2022). Brassicas (e.g., mustards) often produce substantial biomass, whereas annual legumes (e.g., peas) can provide short-term nitrogen (N) inputs. Perennials (e.g., fescues and clovers) establish more slowly in year 1, but form persistent cover that reduces erosion and can improve soil structure via extensive root systems (Abad et al., 2021a). They are also associated with reduced vine vegetative growth, potentially lowering canopy-management needs (leaf removal and hedging), but they require mowing or other control to prevent excess competition (Vanden Heuvel et al., 2021).

Selecting optimal cover crop species for vineyards requires balancing vine performance with ecosystem services. The literature does not explicitly define a unified set of vineyard cover-crop selection criteria; however, our review and synthesis of prior studies (García-Díaz et al., 2018; Abad et al., 2021a; Liebhard et al., 2024; Sharifi et al., 2024) identify a core set of agronomic, ecological, and operational criteria, including i) biomass production (organic-matter inputs and weed suppression); ii) ground-cover percentage (erosion and weed control); iii) low interference with the fruiting zone; iv) low invasiveness/persistence risk; v) pest/disease interactions (e.g., brassicas—flea beetles/aphids/wireworms; vetch—nematodes); vi) drought tolerance for semi-arid, irrigated systems; vii) winter hardiness for overwintering/perennials, especially in cool climates; and viii) traffic/machinery tolerance for inter-row use. Because these criteria often conflict (e.g., high biomass *vs*. vine competition and mowing burden), a multicriteria decision-making framework is warranted to evaluate alternatives comprehensively (Ramírez-García et al., 2015). Furthermore, conventional cover crop selection guides and decision-support tools offer only partial assistance in this complex decision (Liebhard et al., 2024). While there are web-based decision tools (e.g., the Midwest Cover Crop Council and Northeast Cover Crop Council interactive selectors), these are mostly tailored to annual field crop rotations and do not fully account for the perennial cropping context of vineyards (Abad et al., 2021b; Northeast Cover Crops Council, 2021; García et al., 2024; Liebhard et al., 2024; Midwest Cover Crops Council, 2025). They often lack region-specific data and considerations such as vine-row *vs*. inter-row placement of cover crops (Karl et al., 2016; Vanden Heuvel et al., 2021). This knowledge gap is compounded by the limited research specifically focused on cover cropping in vineyards; much of the guidance is extrapolated from other systems or greenhouse trials.

To address these complexities, a multicriteria decision analysis (MCDA) framework was applied to vineyard cover-crop selection, integrating quantitative data, literature information, and expert judgment (Velasquez and Hester, 2013; Taherdoost, 2023). Specifically, the Analytic Hierarchy Process (AHP) structures criteria and derives weights from expert pairwise comparisons (Saaty, 1980, 1987), and Technique for Order Preference by Similarity to Ideal Solution (TOPSIS) ranks species relative to an ideal solution (Hwang and Yoon, 1981), a hybrid used effectively in agricultural suitability studies (Zoma et al., 2023; Sathiyamurthi et al., 2024). Building on the greenhouse screening of 23 species by Sharifi et al. (2024), evaluation was extended to commercial vineyards to i) test the establishment and performance of annual and perennial species in in-row and inter-row zones, ii) identify and weight vineyard-specific criteria from literature and field observations, and iii) generate a multicriteria ranking using AHP–TOPSIS to produce a grower-oriented decision-support tool for cover crop selection in Okanagan Valley, Canada, vineyards. We hypothesized that this hybrid MCDA would discriminate among options and surface species that maximize agronomic benefits (e.g., biomass and ground cover) while minimizing risks (e.g., invasiveness and vine competition), thereby improving on generic tools with vineyard-specific data and priorities.

## 2 Material and methods

### 2.1 Study sites and experimental design

Field experiments were conducted in 2019 at two certified organic vineyards in the Okanagan Valley of British Columbia (BC), Canada. The Okanagan Valley has a semi-arid climate with cool winters, warm summers, and low annual precipitation (~344 mm year^−1^), necessitating irrigation for crop production (Environment and Climate Change Canada, 2025). The two experimental sites were Vineyard 1, Covert Farm Family Estate (CFF Vineyard; Oliver, BC), which is a 13-year-old Merlot (*Vitis vinifera*) block located in the southern Okanagan (49°14′39.8″ N, 119°32′42.7″ W, elevation ~380 m). The site experiences hot, dry summers and has soils classified as loamy sand. Vineyard 2, Kalala Organic Estate Winery (KOW Vineyard; West Kelowna, BC), is a 10-year-old Zweigelt (*V. vinifera*) block in the central Okanagan (49°50′31.2″ N, 119°38′42.0″ W, elevation ~460 m). This site has a slightly cooler climate relative to site 1 (2.8°C average annual) and sandy loam soils. The summary of site characteristics is reported in Lin et al. (2024) (**Supplementary Data Tables S1**, **S2**).

At each vineyard, a randomized complete block design (RCBD) was used with five replicated plots per treatment. Cover crop treatments were categorized by their intended vineyard floor position into in-row (under-vine) and inter-row (alley) groups. Nine in-row treatments were evaluated as under-trellis cover crops, with each plot spanning the length of five to seven consecutive vines (in-row spacing ≈ 1.2 m). The in-row plot was 1 m wide, extending 50 cm on each side of the vine row, and 6–8 m long. Fifteen inter-row treatments were evaluated with each plot covering the full width of one inter-row (spacing = 2.4 to 2.7 m) and matching the in-row plots in length. Cover crop species and mixtures were selected based on a previous greenhouse study (Sharifi et al., 2024), literature recommendations for vineyards in similar climates (Olmstead et al., 2001), and input from local experts. The nine in-row treatments include seven annual or biennial species, i.e., common buckwheat (*Fagopyrum esculentum*), field pea (*Pisum sativum*), white mustard (*Sinapis alba*), phacelia (*Phacelia tanacetifolia*), spring lentil (*Lens culinaris*), purple top turnip (*Brassica rapa* subsp. rapa), and Winfred brassica (*Brassica napus* cv. ‘Winfred’); and two perennials, i.e., buffalo grass (*Bouteloua dactyloides*) and Ladino white clover (*Trifolium repens* cv. ‘Crescendo’).

The 15 inter-row cover crop treatments were grouped into annual and perennial categories. Annual treatments included legumes, cereals, and broadleaf species: field pea + cereal rye (*Secale cereale*), berseem clover (*Trifolium alexandrinum*), crimson clover (*Trifolium incarnatum*), alsike clover (*Trifolium hybridum*), persian clover (*Trifolium resupinatum*), balansa clover (*Trifolium michelianum*), Indian ricegrass (*Oryzopsis hymenoides*) + buckwheat, and hairy vetch (*Vicia villosa*, Roth) + cereal rye. Perennial treatments include perennial ryegrass + tillage radish (*Lolium perenne* + *Raphanus sativus* var. *longipinnatus*), birdsfoot trefoil + western wheatgrass (*Lotus corniculatus* + *Pascopyrum smithii*), a fescue mix (tall, red, and sheep fescue; *Festuca arundinacea*, *Festuca rubra*, and *Festuca ovina*), crested wheatgrass + pubescent wheatgrass (*Agropyron cristatum* + *Thinopyrum intermedium*), blue grama (*Bouteloua gracilis*), western wheatgrass, and Canada bluegrass (*Poa compressa*). All cover crops were sown in late spring 2019 after frost risk: in-row in late May (post-budbreak) and inter-row in early June. The seeding rates followed supplier and literature guidance, adjusted for pure live seed and mix proportions (**Table 1**). In-row plots were hoed for weed removal and to prepare the seedbed, then hand-seeded and lightly raked in; inter-row plots were drill-seeded (Land Pride compact no-till; Division of Great Plains Manufacturing, Salina, KS, USA) in CFF Vineyard, cultivated using a rototiller, and seeded using a grass seeder with roller in the back (Brillion Sure-Stand SS-5; Landoll, Marysville, KS, USA) at the KOW Vineyard. A dual irrigation system was in place at both vineyards: drip emitters in the vine row for vine irrigation and microsprinklers or under-canopy sprinklers to irrigate in-row cover crops. Under-canopy sprinklers were scheduled for 4-h sets once per week in June and September (cooler conditions) and twice per week in July–August when temperatures approached 30°C. This ensured that cover crops received sufficient moisture independent of the vines’ drip system. Both vineyards were managed with organic-approved practices for fertilization and pest control, and no synthetic chemicals were applied. Cover crop plots were mowed when vegetation reached ~30-cm height or ~30% flowering to simulate grower floor management practices and prevent reseeding of annuals. Mowing dates varied by species growth rates, but generally occurred when needed between mid-summer to early-fall. Notably, some fast-growing species (e.g., buckwheat and mustard) reached 30% flowering quite early (~40 days after planting), whereas slower perennials were not mowed until later. In-row and inter-row cover crops received neither fertilization nor supplemental management interventions during the study period.

**Table 1:** Seeding rates for in-row and inter-row cover crops in the two study vineyards.

Species	Scientific name	Variety	Family/type	Seeding rate (kg ha^−1^)^1^	
				CFF vineyard	KOW vineyard
In-row					
Winfred brassica	*Brassica napus* L.	Winfred	Brassicaceae/forbs	4.5	4.5
Buckwheat	*Fagopyrum esculentum* Moench	Common	Brassicaceae/forbs	59	59
Field pea	*Pisum sativum* L.	Horizon	Fabaceae/legume	56	56
White mustard	*Sinapis alba* L.	Common	Brassicaceae/forbs	6.7	6.7
Phacelia	*Phacelia tanacetifolia* Benth	Common	Boraginaceae/forbs	9.0	9.0
Purple top turnip	*Brassica rapa* subsp. *rapa*	Common	Brassicaceae/forbs	5.0	5.0
Spring lentil	*Lens culinaris* Medik.	Spring	Fabaceae/legume	34	34
Ladino white clover	*Trifolium repens* L.	Crescendo	Fabaceae/legume	4.5	4.5
Buffalo grass	*Bouteloua dactyloides* (Nutt.) J.T.Columbus	Common	Poaceae/grass	49	49
Inter-row					
Balansa clover	*Trifolium michelianum* Savi	Common	Fabaceae/legume	9	9
Berseem clover	*Trifolium alexandrinum* L.	Common	Fabaceae/legume	30	36
Persian clover	*Trifolium resupinatum* L.	Common	Fabaceae/legume	6	6
Crimson clover	*Trifolium incarnatum* L.	Flame	Fabaceae/legume	40	33
Alsike clover	*Trifolium hybridum* L.	Common	Fabaceae/legume	8	8
Hairy vetch + Cereal rye	*Vicia villosa* Roth + *Secale cereale* L.	Common + Yankee	Fabaceae/legume + Poaceae/grass	50 + 101	34 + 95
Field pea + Cereal rye	*Pisum sativum* L. + *Secale cereale* L.	Horizon + Yankee	Fabaceae/legume + Poaceae/grass	94 + 101	94 + 95
Crested wheatgrass + Pubescent wheatgrass	*Agropyron cristatum* (L.) Gaertn. + *Thinopyrum intermedium* (Host) Barkworth & D.R.Dewey	Fairway	Poaceae/grass	23 + 25	19 + 24
Indian ricegrass + Buckwheat	*Oryzopsis hymenoides* (Roem. & Schult.) Ricker ex Piper + *Fagopyrum esculentum* Moench	Common	Poaceae/grass + Brassicaceae/forbs	30 + 68	30 + 40
Blue grama	*Bouteloua gracilis* (Kunth) Lag. ex Griffiths	Common	Poaceae/grass	13	11
Western wheatgrass	*Pascopyrum smithii* (Rydb.) Á.Löve	Common	Poaceae/grass	45	33
Tall fescue + Red fescue + Sheep fescue	*Festuca arundinacea* Schreb. + *Festuca rubra* L. + *Festuca ovina* L.	Kentucky 32 + Boreal + Covar	Poaceae/grass	32 + 19 + 6	23 + 10 + 3
Perennial ryegrass + Tillage radish	*Lolium perenne* L. + *Raphanus sativus* L. var. *longipinnatus* (L.H.Bailey) Kitam.	Pennington + Aerifi	Poaceae/grass + Brassicaceae/forbs	40 + 31	40 + 8
Canada blue grass	*Poa compressa* L.	Common	Poaceae/grass	4	4
Birdsfoot trefoil + Western wheatgrass	*Lotus corniculatus* L. + *Pascopyrum smithii* (Rydb.) Á.Löve	Wellington + common	Fabaceae/legume + Poaceae/grass	9 + 45	9 + 17

^1^ Inter-row seeding rates differed between vineyards because different equipment was used; the drill seeder was more accurate and delivered rates closer to the target. In-row plots were seeded by hand.

### 2.2 Cover crop performance and criterion measurements

Multiple cover crop criteria were recorded during the growing season; however, these variables were narrowed down to the most effective parameters with minimum overlap based on literature (Olmstead et al., 2001; Sharifi et al., 2024) and expert opinion. At each site, assessments were performed just before each mowing or at a similar growth stage for all plots:

- Canopy coverage: The percentage of ground covered by the cover crop *vs*. weeds was estimated using a quadrat method. Two 0.25-m^2^ quadrats were placed randomly in each plot, and the canopy cover of a) the sown cover crop and b) weeds or volunteer plants was visually estimated (Daubenmire cover class method; Daubenmire, 1959).
- Aboveground biomass: Following the coverage estimation, all vegetation within each quadrat was clipped at 2.5 cm above the ground. The clippings were separated into cover crop biomass *vs*. weed biomass, then dried at 60°C to constant weight (~48 h), and weighed. Dry matter is expressed in kg ha^−1^ (extrapolated from quadrat weights). The total cover crop dry matter produced over the growing season in each plot was computed by summing biomass from multiple cuts if a plot was mowed more than once.
- Interference with fruiting zone: In the in-row plots, we observed whether the cover crop plants reached up into the vine canopy’s fruiting zone (approximately 40–50 cm above ground for these low-trained vines). We noted any instance of cover crop tendrils or stems intertwining with grape clusters or climbing the vine trunk qualitatively for each species.
- Invasiveness potential: Traits such as prolific seed production and evidence of self-seeding were recorded and used to rank each species’ invasiveness risk as high, medium, or low.
- Pest and disease incidence: Plots were scouted for any notable pest damage or disease on the cover crops. These observations were documented as either presence/absence or minor *vs*. major damage.
- Drought tolerance: Although irrigation was provided, the summer climate is dry and hot in the Okanagan Valley region of Canada, so it was noted how well each cover crop maintained vigor between irrigation events. Species that visibly wilted or senesced quickly under limited moisture were rated lower in drought tolerance, whereas those that remained green were rated higher.
- Winter hardiness: For perennial and winter-annual species, their ability to survive the winter was assessed. Winter minimum temperatures in these areas can reach approximately –15°C to –20°C. Hardiness rating was assigned based on the known hardiness of each species (USDA, 2012).
- Traffic tolerance: In the inter-row plots, which were driven on during routine vineyard operations (mowing, spraying, hedging, etc.), any damage to cover crops from wheel traffic was qualitatively recorded. This criterion was specifically considered only for inter-row covers, as in-row areas do not experience wheel traffic.

The above measurements provided the data needed to score each cover crop against the selection criteria. To reduce redundancy, some highly correlated measurements were excluded from the final criterion list. For example, total dry matter and percent cover are related; both were retained initially but weighted accordingly to avoid double-counting.

### 2.3 Multicriteria decision analysis framework

**Figure 1:**
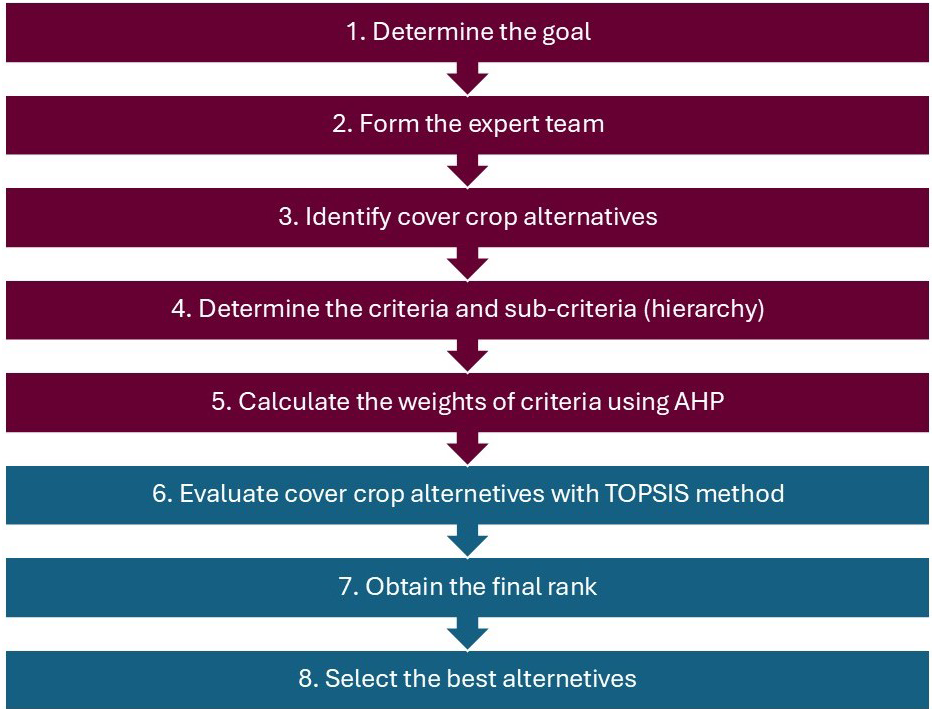
Flowchart outlining an eight-step process for selecting cover crops using combined Analytic Hierarchy Process (AHP) and Technique for Order Preference by Similarity to Ideal Solution (TOPSIS) models.

A hybrid AHP–TOPSIS approach was used to evaluate and rank cover crop species for use in irrigated vineyards in the Okanagan Valley, Canada (**Figure 1**). First, AHP was used to determine the weights of each criterion. These weights were then applied in the TOPSIS method to select the best alternatives.

#### 2.3.1 AHP framework for decision hierarchy and criterion weighting

The AHP is a structured decision-making method that decomposes complex problems into hierarchically organized criteria. This framework facilitates systematic comparison of alternatives and is particularly suitable when subjectivity is involved. According to Saaty (1990, 2008) and Saaty and Vargas (2001), the methodology involves three main steps:

1. Problem definition and criterion identification: Clearly define the decision problem and determine the relevant criteria and sub-criteria, ensuring consistency and logical relationships among factors (Lee et al., 2012).
2. Structuring the decision hierarchy: Arrange the decision framework hierarchically, placing the overall goal at the top, followed by main criteria, sub-criteria, and alternatives at subsequent levels.
3. Constructing the pairwise comparison matrix: Compare criteria pairwise using a fundamental comparison scale. Each n × n matrix includes reciprocal values, with the number of required comparisons calculated as n(n − 1)/2 (Lee et al., 2012) (Equations 1, 2).
4. Synthesis of priorities and consistency check: Derive global priorities by calculating the principal right eigenvector and the largest eigenvalue of the comparison matrix (Equation 3). This step synthesizes local weights into overall priorities and includes a consistency check to ensure logical coherence of judgments. Matrix A = (a_ij_) is said to be consistent if a_ij_ · a_jk_ = a_ik_ for all i, j, k, and its principal eigenvalue (λ_max_) is equal to n.

The general eigenvalue formulation is:

(1) $$[\begin{array}{cccc} 1 & {\text{w}}_{1}/{\text{w}}_{2} & \cdots & {\text{w}}_{1}/{\text{w}}_{n}\\ {\text{w}}_{2}/{\text{w}}_{1} & 1 & \cdots & {\text{w}}_{2}/{\text{w}}_{n}\\ \vdots & \vdots & \vdots & \vdots \\ {\text{w}}_{n}/{\text{w}}_{1} & {\text{w}}_{n}/{\text{w}}_{2} & \cdots & 1 \end{array}][\begin{array}{c} {\text{w}}_{1}\\ {\text{w}}_{2}\\ \vdots \\ {\text{w}}_{n} \end{array}]=\text{nw}$$

**-** Weight vector w is an n × 1 column vector of priority weights:


$$\text{w}=[\begin{array}{c} {\text{w}}_{1}\\ {\text{w}}_{2}\\ \vdots \\ {\text{w}}_{n} \end{array}]$$

Each w_i_ represents the relative importance (priority) of criterion i.

**-** Pairwise comparison element a_ij_ represents the relative importance of element iii compared to element j.

(2) $${\text{a}}_{\text{ij}}={\text{w}}_{\text{i}}/{\text{w}}_{\text{j}},\text{ i},\text{j}=1,2,\ldots ,\text{n}$$

where w_i_ and w_j_ are the weights of criteria i and j.

**-** Matrix–vector multiplication A_w_ is the product of the pairwise comparison matrix A and the weight vector w:

(3) $${\text{A}}_{\text{w}}={\text{λ}}_{\text{max}}\text{w}$$

This is the eigenvalue equation, where λ_max_ is the principal (largest) eigenvalue.

Principal eigenvalue λ_max_ is a scalar that satisfies the eigenvalue equation above. In the case of perfect consistency, λ_max_ = n.

Consistency index (CI) is a measure of how consistent the comparisons are. The closer CI is to 0, the more consistent the judgments (Equation 4).

(4) $$\text{CI}=({\text{λ}}_{\text{max}}-\text{n})/(\text{n}-1)$$

To ensure the reliability of the results, the consistency of the matrix is evaluated using the consistency ratio (CR). The CI is compared with the average random consistency index (RI) to obtain the CR (Equation 5; **Table 2**). Acceptable values of CR must be less than 0.1 (Saaty, 1990). If the CR is significantly small, the estimate of the weights is accepted. However, if the CR value is too high, it indicates that the experts’ judgments are inconsistent, necessitating a review of the pairwise comparisons (Lee et al., 2012; Saaty, 1980).

(5) $$\text{CR}=\frac{CI}{RI}$$

**Table 2:** Average random consistency index (RI) for pairwise comparison matrices of size 1–10, used to evaluate consistency in AHP decision models.

n	1	2	3	4	5	6	7	8	9	10
Random consistency index (RI)	0	0	0.52	0.89	1.11	1.25	1.35	1.40	1.45	1.49

This process allows decision-makers to systematically evaluate each criterion’s relative importance and, subsequently, synthesize the results to identify the most appropriate decision or alternative.

#### 2.3.2 TOPSIS ranking of cover crop species

The TOPSIS is a widely used multicriteria decision-making method that ranks alternatives based on their relative closeness to an ideal solution (Zoma et al., 2023). One of its advantages is that it avoids the need for pairwise comparisons, making it simpler than methods such as AHP. The procedure (Tsaur, 2011) involves the following steps:

1. Construct the decision matrix: Define the set of alternatives A_1_, A_2_, …, A_n_ and criteria C_1_, C_2_, …, C_m_, where x_ij_ is the performance value of alternative A_i_ with respect to criterion C_j_ (Equation 6).

(6) $$\begin{array}{c} \text{m Criteria}\\ {\text{C}}_{1}\;{\text{C}}_{2}\;\cdots \;{\text{C}}_{\text{j}}\;\cdots \;{\text{C}}_{\text{m}}\\ \text{X}=[\begin{array}{cccccc} x_{11} & x_{12} & \cdots & x_{1j} & \cdots & x_{1m}\\ x_{21} & x_{22} & \cdots & x_{2j} & \cdots & x_{2m}\\ \vdots & \vdots & \vdots & \vdots & \vdots & \vdots \\ x_{i1} & x_{i2} & \cdots & x_{ij} & \cdots & x_{im}\\ \vdots & \vdots & \vdots & \vdots & \vdots & \vdots \\ x_{n1} & x_{n2} & \cdots & x_{nj} & \cdots & x_{nm} \end{array}]\begin{array}{c} A_{1}\\ A_{2}\\ \vdots \\ A_{i}\\ \vdots \\ A_{n} \end{array}\}\text{n} \end{array}$$

2. The relative weight vector for the criteria is W = (w_1_, w_2_, …,w_j_, …,w_m_), where w_j_ represents the weight of the *j*th attribute. It represents the relative importance of criteria, and the sum of the weights equals 1.


$$\sum_{j=1}^{m}w_{j}=1$$

3. Normalize the decision matrix: To ensure that the criteria are comparable, the decision matrix is normalized using (Equation 7):

(7) $$r_{ij}=\;\frac{x_{ij}}{\sqrt{\sum_{i=1}^{n}x_{ij}^{2}}}\text{ i}=1,2,3,\ldots ,\text{n j}=1,2,3,\ldots ,\text{m}$$

4. Weighted normalized decision matrix: Each normalized value is multiplied by the corresponding weight (Equation 8).

(8) $${\text{v}}_{\text{ij}}={\text{w}}_{\text{j}}{\text{r}}_{\text{ij}}\text{ i}=1,2,3,\ldots ,\text{n j}=1,2,3,\ldots ,\text{m}$$

5. Identify the positive ideal solution (PIS) and negative ideal solution (NIS): The PIS and NIS are identified based on the beneficial or non-beneficial nature of each criterion (Equations 9, 10). The PIS is the best possible value for each criterion, while the NIS is the worst. These are expressed as

(9) $$\text{PIS}={\text{A}}^{*}=\{{\text{v}}_{1}^{*},{\text{v}}_{2}^{*},\ldots ,{\text{v}}_{\text{m}}^{*}\}=\{(\underset{i}{\max}\;\;{\text{v}}_{\text{ij}}|\text{j }є\;{\text{Ω}}_{\text{b}}),(\underset{i}{m\text{in}}\;{\text{v}}_{\text{ij}}|\text{j }є\;{\text{Ω}}_{\text{c}})\}$$

(10) $$\text{NIS}={\text{A}}^{-}=\{{\text{v}}_{1}^{-},{\text{v}}_{2}^{-},\ldots ,{\text{v}}_{\text{m}}^{-}\}=\{(\underset{i}{\min}\;\;{\text{v}}_{\text{ij}}|\text{j }є\;{\text{Ω}}_{\text{b}}),(\underset{i}{\text{max}}\;{\text{v}}_{\text{ij}}|\text{j }є\;{\text{Ω}}_{\text{c}})\}$$

where Ω_b_ is the set of criteria to be maximized (beneficial criteria) and Ω_c_ is the set of criteria to be minimized (non-beneficial criteria).

Determine the separation measures (Euclidean distance): The Euclidean distance of each alternative from the PIS and NIS is then calculated separately as follows, where d_i_^*^ is the separation measure from the PIS and d_i_^−^ is the separation measure from the NIS (Equations 11, 12).

(11) $$d_{i}^{*}\;=\;\sqrt{\sum_{j=1}^{m}(v_{ij}}-\;v_{j}^{*}{)}^{2}\;\;,\text{ i}=1,2,3,\ldots ,\text{n}$$

(12) $$d_{i}^{-}\;=\;\sqrt{\sum_{j=1}^{m}(v_{ij}}-\;v_{j}^{-}{)}^{2}\;\;,\text{ i}=1,2,3,\ldots ,\text{n}$$

Calculate the relative closeness to the ideal solution: The relative closeness (RC_i_) indicates how close an alternative is to the ideal solution, with values closer to 1 indicating better performance (Equation 13).

(13) $$\begin{array}{l} {\text{RC}}_{\text{i}}=\frac{d_{i}^{-}}{d_{i\;}^{*}+d_{i}^{-}},\text{ i}=1,2,3,\ldots ,\text{n}\\ {\text{RC}}_{\text{i}}є[0,1] \end{array}$$

6. Rank the alternatives: Finally, the alternatives are ranked in descending order of RC_i_ values. The higher the relative closeness to the ideal solution, the better the rank of the alternative.

In the current study, the workflow for using the hybrid AHP–TOPSIS method was as follows (**Figure 1**):

1. Define the problem and hierarchy (AHP): Establish the goal, criteria, and alternatives.
2. Perform pairwise comparisons (AHP): Conduct pairwise comparisons for criteria to derive their relative weights. Use AHP to calculate the weights and ensure consistency.
3. Evaluate alternatives (TOPSIS): Use the weights from AHP in the TOPSIS method to evaluate and rank the cover crop alternatives.

The overall objective of this study was to select the optimal cover crop species for each region. Cover crops were categorized into four groups based on spatial position and life cycle: in-row annual, in-row perennial, inter-row annual, and inter-row perennial (**Table 3**). The decision hierarchy comprised three levels. Level 1 (Criteria Groups) initially included three groups of evaluation criteria: seed-related, physiological, and biotic and abiotic stresses. Seed-related criteria (seed availability and cost per hectare) were later excluded because they reflect market-driven conditions that vary across regions and over time and therefore do not represent stable, crop-intrinsic attributes. While seed factors may matter for region-specific or farm-level choices, their exclusion ensured that the ranking framework remained generalizable and biologically grounded. Level 2 (Criteria) encompassed physiological criteria [total cover-crop dry biomass, ground coverage, interference with the grape’s fruiting zone (for in-row only), and the risk of being invasive] and response to biotic and abiotic stresses criteria [sensitivity to pests, drought tolerance, winter hardiness, and traffic tolerance (for inter-row only)] (**Table 3**). Level 3 (Alternatives) comprised the candidate cover crop species evaluated within their respective categories.

**Table 3:** Hierarchical structure to evaluate cover crop alternatives for North and South Okanagan Valley using AHP method.

Positioning—growth cycle	Level 1: main selection criteria	Level 2: sub-criteria	Level 3: cover crop alternatives
In-row annuals	1. Physiological criteria	1.1. Total dry biomass 1.2. Ground coverage 1.3. Interfere with grape’s fruiting zone 1.4. Risk of being invasive	Winfred Brassica Buckwheat Field Pea White Mustard Phacelia Turnip Spring Lentil
	2. Response to biotic and abiotic stress criteria	2.1. Sensitivity to pests 2.2. Drought tolerance 2.3. Winter hardiness	
In-rows Perennials			Crescendo Ladino White Clover
Inter-row annuals	1. Physiological criteria	1.1. Total dry biomass 1.2. Ground coverage 1.3. Risk of being invasive	Balansa Clover Berseem clover Persian clover Crimson clover Alsike clover Hairy vetch + Cereal rye Field pea + Cereal rye
	2. Response to biotic and abiotic stress criteria	2.1. Sensitivity to pests 2.2. Drought tolerance 2.3. Winter hardiness 2.4. Traffic tolerance	
Inter-rows perennials			Crested wheatgrass + Pubescent wheatgrass Indian ricegrass + Buckwheat Blue grama Western wheatgrass Tall fescue + Red fescue + Sheep fescue Perennial ryegrass + Tillage radish Canada blue grass Birdsfoot trefoil + Western wheatgrass

AHP, Analytic Hierarchy Process.

Using the AHP methodology, the relative importance of the selection criteria was determined through pairwise comparisons within each decision category (**Table 4**, **Supplementary Data Tables S4**, **S5**). This process involved input from experts (including researchers, vineyard managers, and growers), who evaluated the criteria based on professional judgment and field observations. Saaty’s 1–9 scale was used for the comparisons, where 1 indicates equal importance and 9 indicates extreme preference of one criterion over another (Saaty, 1980). The resulting judgments were compiled into a pairwise comparison matrix for each category. To ensure the reliability of the expert input, consistency was assessed by calculating the CR for each matrix. All CR values were below the accepted threshold of 0.10, indicating that the expert judgments were consistent.

**Table 4:** The fundamental scale of pair-wise comparison for AHP.

Intensity of importance	Definition	Explanation
1	Equal importance	Equal importance: two activities have equal contribution to the objective
3	Moderate importance	Experience and judgment slightly favor one activity over another
5	Strong importance	Experience and judgment strongly favor one activity over another
7	Very strong on demonstrated importance	An activity is favored very strongly over another
9	Extreme importance	The evidence favoring one activity over another is of the highest possible order of affirmation
2, 4, 6, 8	For compromise between the above values	Sometimes, one needs to interpolate a compromise judgment numerically

AHP, Analytic Hierarchy Process.

The AHP procedure was then applied to calculate the weight vector for each set of criteria. Specifically, the relative weights were derived as the normalized principal eigenvector of each pairwise comparison matrix. For in-row alternatives, the “traffic tolerance” criterion was not included in the weight calculation because it is only relevant for inter-row conditions, whereas for inter-row conditions, “interference with fruiting zone” was not included (**Table 3**). After determining the criterion weights, the TOPSIS method was applied to rank the cover crop options in each category. For each category (e.g., in-row annuals), a decision matrix was created using field data (i.e., total dry biomass and ground coverage) or field observations and literature data (i.e., for the rest of the criteria) for cover crop alternatives. Quantitative criteria like total dry biomass and ground cover were directly included, while qualitative criteria such as invasiveness or pest susceptibility were scored numerically. Each criterion is either beneficial (where higher values are preferred) or non-beneficial (where lower values are preferred) (**Supplementary Data Table S4**). All values were scaled appropriately so that higher scores consistently reflected better performance across all criteria. Using RC_i_ values, a clear indicator of how well each cover crop meets the multicriteria objective relative to an ideal solution was obtained. An RC_i_ value of 1 represents a hypothetical cover crop that perfectly satisfies all criteria, whereas an RC_i_ near 0 indicates a poor-performing alternative. In practice, RC_i_ values typically fall between these extremes, enabling effective differentiation between strong, moderate, and weak candidates.

## 3 Results

### 3.1 Cover crop field performance and input criteria

The cover crop species exhibited a wide range of agronomic, ecological, and operational criteria, which in turn influenced their evaluation criteria (**Tables 5**, **6**). In the in-row setting, fast-growing annuals (e.g., brassicas and turnips) generally delivered rapid canopy closure and strong short-term suppression, but this came with trade-offs (**Table 5**). Winfred brassica suffered noticeable aphid (*Lipaphis* spp.) infestations on its foliage, and turnip roots appeared to attract wireworms (*Limonius* spp.), evidenced by damage holes; however, the grapevine was not affected by any of these pests. Buckwheat provided quick cover, yet was the most drought-sensitive, and carried a higher self-seeding concern (**Table 5**). Field pea tended to intrude toward the fruiting zone under vigorous growth, whereas spring lentil, a shorter vine-legume, established rapidly and well in-row, did not climb, and efficiently competed with weeds; however, it senesced mid-summer (**Table 5**). Perennial options contrasted sharply: Ladino white clover offered a steadier, more resilient in-row choice with better cold tolerance, while buffalo grass remained slow to establish and provided limited first-year utility (**Table 5**). Site effects were evident across species, underscoring that local conditions can tip the balance among these trade-offs.

**Table 5:** Cover crops in-row input data for multi-decision-making model (AHP–TOPSIS) at Covert Family Farm Estate (CFF vineyard) and Kalala Organic Estate Winery (KOW vineyard).

Life cycle	Cover crop species	Dry biomass (kg ha^−1^)^1^		Ground coverage (1–5)^1^		Fruiting zone interference (1–3)	Risk of being invasive (1–3)	Sensitivity to pests (1–3)	Drought tolerance (1–3)	Winter hardiness (1–7)
		CFF Vineyard	KOW Vineyard	CFF Vineyard	KOW Vineyard					
Annual	Winfred brassica	2,860	7,343	2.1	2.6	1	1	2	1	4
	Buckwheat	2,186	1,514	3.6	2.6	2	2	1	1	1
	Field pea	2,113	1,550	2.9	2.8	3	1	2	2	2
	White mustard	994	762	1.7	1.5	2	3	2	1	4
	Phacelia	1,587	847	2.3	1.6	2	1	1	1	1
	Purple top turnip	3,249	3,107	3.9	3.4	1	1	2	1	2
	Spring lentil	1,942	1,917	2.9	2.7	1	1	1	2	2
Perennial	Crescendo Ladino white clover	3,046	3,571	2.1	1.7	1	1	2	2	5
	Buffalo grass	1	157	1.0	1.5	1	1	1	3	3

Ground coverage: 0 = 0%–5%, 1 = 5%–25%, 2 = 25%–50%, 3 = 50%–75%, and 4 = 75%–100%. Fruiting zone interference (GiESCO vineyard floor management guide; Vanden Heuvel et al., 2021): 1 = low interference risk—prostrate or low-growing species, stay beneath fruit zone; 2 = moderate interference risk—taller annuals, may reach lower clusters in vigorous years; and 3, = high interference risk—vining or tall species that regularly intrude into canopy/fruit zone. Risk of being invasive (Weed Risk Assessment Frameworks; Pheloung et al., 1999): 1 = low risk, 2 = medium risk, and 3 = high risk. Pest/disease pressure indices (IPPC, 2019): 1 = low (rarely associated with vineyard pests), 2 = moderate (occasional pest associations), and 3 = high (known reservoir of major vineyard pests/diseases). Drought tolerance (FAO agronomic rating; FAO/IIASA, 2025): 1 = sensitive, 2 = moderately tolerant, and 3 = highly tolerant. Winter hardiness [Royal Horticultural Society (RHS) rating]: 1 = H1c (5°C to 10°C), 2 = H2 (1°C to 5°C), 3 = H3 (−5°C to 1°C), 4 = H4 (−10°C to −5°C), 5 = H5 (−15°C to −10°C), 6 = H6 (−20°C to −15°C), and 7 = H7 (<−20°C).

AHP, Analytic Hierarchy Process; TOPSIS, Technique for Order Preference by Similarity to Ideal Solution.

^1^ Data are average of five replications (n = 5).

**Table 6:** Cover crops inter-row input data for multi-decision-making model (AHP–TOPSIS) Covert Family Farm Estate (CFF Vineyard) and Kalala Organic Estate Winery (KOW Vineyard).

Life cycle	Cover crop species	Dry biomass (kg ha^−1^)^1^		Ground coverage (1–5)^1^		Risk of being invasive (1–3)	Sensitivity to pests (1–3)	Drought tolerance (1–3)	Winter hardiness (1–7)	Tolerance to traffic (1–2)
		CFF Vineyard	KOW Vineyard	CFF Vineyard	KOW Vineyard					
Annual	Balansa clover	3,176	1,263	0.8	1.7	1	2	2	7	1
	Berseem clover	1,022	6,253	0.6	3.9	1	1	1	4	1
	Persian clover	1,316	1,750	1.1	1.7	1	1	1	3	1
	Crimson clover	1,701	1,822	1.4	3.4	1	1	1	3	1
	Alsike clover	2,678	1,678	0.6	1.8	1	2	2	4	1
	Hairy vetch + Cereal rye	7,891	6,512	3.0	2.8	3	2	2	6	2
	Field pea + Cereal rye	4,151	8,579	2.9	3.3	1	2	2	2	2
Perennial	Crested wheatgrass + Pubescent wheatgrass	1,124	1,323	0.7	1.5	1	1	3	6	2
	Indian ricegrass + Buckwheat	2,037	1,668	2.6	3.3	2	1	1	2	1
	Blue grama	491	249	0.8	2	1	1	3	3	2
	Western wheatgrass	592	911	0.6	1.4	2	1	3	6	2
	Tall fescue + Red fescue + Sheep fescue	2,215	1,704	1.0	1.6	1	1	2	6	2
	Perennial ryegrass + Tillage radish	6,679	4,515	2.6	3.5	1	1	1	4	2
	Canada blue grass	455	368	0.6	1	2	1	2	5	2
	Birdsfoot trefoil + Western wheatgrass	891	1,140	0.7	2	2	1	2	6	2

Ground coverage: 0 = 0%–5%, 1 = 5%–25%, 2 = 25%–50%, 3 = 50%–75%, and 4 = 75%–100%. Fruiting zone interference (GiESCO vineyard floor management guide; Vanden Heuvel et al., 2021): 1 = low interference risk—prostrate or low-growing species, stay beneath fruit zone; 2 = moderate interference risk—taller annuals, may reach lower clusters in vigorous years; and 3 = high interference risk—vining or tall species that regularly intrude into canopy/fruit zone. Risk of being invasive (Weed Risk Assessment Frameworks; Pheloung et al., 1999): 1 = low risk, 2 = medium risk, and 3 = high risk. Pest/disease pressure indices (IPPC, 2019): 1 = low (rarely associated with vineyard pests), 2 = moderate (occasional pest associations), and 3 = high (known reservoir of major vineyard pests/diseases). Drought tolerance (FAO agronomic rating; FAO/IIASA, 2025): 1 = sensitive, 2 = moderately tolerant, and 3 = highly tolerant. Winter hardiness [Royal Horticultural Society (RHS) rating]: 1 = H1c (5°C to 10°C), 2 = H2 (1°C to 5°C), 3 = H3 (−5°C to 1°C), 4 = H4 (−10°C to −5°C), 5 = H5 (−15°C to −10°C), 6 = H6 (−20°C to −15°C), and 7 = H7 (<−20°C).

AHP, Analytic Hierarchy Process; TOPSIS, Technique for Order Preference by Similarity to Ideal Solution.

^1^ Data are average of five replications (n = 5).

Inter-row mixtures containing cereal rye were the most effective options, delivering consistently high biomass, rapid ground cover, and superior wheel-traffic tolerance (**Table 6**). Adding hairy vetch further increased early biomass and competitiveness but elevated self-seeding and encroachment risk. The pea–cereal rye mix provided a lower-risk alternative with comparably strong performance. Cereal rye-based stands suppressed weeds strongly, consistent with rapid canopy closure and possible allelopathic effects. Perennial grass blends (fescues, wheatgrasses, and perennial ryegrass + tillage radish) offered excellent winter hardiness and traffic tolerance but slower establishment, requiring a longer horizon to realize benefits (**Table 6**). Native/fine-textured grasses (e.g., blue grama and Canada bluegrass) established conservatively, prioritizing durability and low stature over rapid cover. Indian ricegrass failed to establish under field conditions, and buffalo grass was hard to establish and cost-prohibitive at the time of study. Birdsfoot trefoil exhibited poor first-season competitiveness despite drought tolerance. Annual clovers were site-responsive, performing best under cooler, moister conditions (KOW Vineyards) but were unsuitable for high-traffic alleys; among these, crimson, berseem, and alsike clovers established most reliably (**Table 6**). White mustard and buckwheat posed invasiveness/seed-set risks if not timely mowed; buckwheat was drought-sensitive, and both species grew tall (~60 cm), risking fruit-zone interference in in-row contexts. Collectively, these results underscore that species choice and termination timing must be calibrated to site water status, vine vigor, and traffic intensity.

### 3.2 AHP criterion weight summary

The AHP analysis of expert judgment and literature data, as introduced above, yielded a set of weights reflecting criterion priorities (**Table 7**). The risk of being invasive was the top-ranked criterion across the board, especially for inter-row covers (~0.60) and also high for in-row (0.43). The second-highest criterion for in-row covers was interference with the fruiting zone (0.31 weight). In inter-rows, ground coverage was the next important criterion (0.13–0.15), signifying the potential to prevent erosion and suppress weeds. Sensitivity to pests had a moderate weight (~0.11–0.13) in both contexts, reflecting concern that certain cover crops may harbor pests. Other criteria, such as drought tolerance, winter hardiness, and traffic tolerance, were comparatively low-weight (<0.05 each in most cases), indicating that while these traits are considered, they were not the main drivers in the decision relative to the others. Biomass production received low weighting (0.03–0.07), indicating that moderate, non-interfering biomass was preferred over maximum biomass that could increase weediness or management demands in the vineyard production system.

**Table 7:** AHP-derived importance weights for cover crop selection criteria (separate analyses for in-row *vs*. inter-row; criteria not applicable in a given context are marked N/A).

Criteria	In-row^1^ weights	Inter-row weights	
	Annual/perennial	Annual	Perennial
Cover crop dry biomass	0.034	0.067	0.070
Cover crop ground coverage	0.060	0.125	0.148
Interference with grape’s fruiting zone	0.307	N/A	N/A
Risk of being invasive (spread)	0.432	0.596	0.572
Sensitivity to pests (diseases and insects)	0.129	0.115	0.123
Drought tolerance	0.026	0.013	0.033
Winter hardiness (overwinter survival)	0.011	0.039	0.011
Tolerance to traffic (wheel compaction)	N/A	0.045	0.044

The weight values were defined based on expert opinion and logic. In the AHP hierarchy, in-row annual and in-row perennial cover crops shared the same criterion weights, as the criteria and their relative importance were considered equivalent for both life cycles under vines. Inter-row annual *vs*. perennial covers were evaluated in separate AHP runs, yielding slight differences in weights—e.g., winter hardiness carries more weight for annual cover crops in the inter-row than for perennials because a winter-killed annual’s residue could be beneficial, whereas perennials are expected to survive.

AHP, Analytic Hierarchy Process.

^1^ The consistency ratio (CR) indicates the consistency of the pairwise comparisons. The CR ranged from 0.06 to 0.09 for the criteria. A CR value of 0.10 or less is generally considered acceptable, indicating a high level of consistency in the judgments.

### 3.3 Cover crops ranking at Covert Family Farm Estate

Using the level 2 criterion data and criterion weights, the TOPSIS procedure was executed to rank the cover crop alternatives at CFF Vineyard (**Tables 5**, **6**). Higher RC_i_ values (closer to 1) indicate a better overall performance relative to the ideal. At CFF Vineyard, the in-row annual rankings placed spring lentil at the top with RC_i_ of 0.96. Purple top turnip edged slightly above Winfred brassica (RC_i_ = 0.89 *vs*. 0.88), but both remained high performers (**Table 8**). Ladino white clover was a top in-row perennial species (RC_i_ = 0.86) at CFF Vineyard, while buffalo grass showed much lower RC_i_. Phacelia and field pea ranked intermediate, while buckwheat and mustard had the lowest RC_i_ values (0.23–0.51), indicating poor performers.

**Table 8:** Cover crop screening and ranking at Covert Family Farm Estate (MCDA AHP–TOPSIS results).

In-row (annual species)	RC_i_	In-row (perennial species)	RC_i_
Spring lentil	0.96	Ladino white clover	0.86
Purple top turnip	0.89	Buffalo grass	0.14
Winfred brassica	0.88	–	–
Phacelia	0.77	–	–
Field pea	0.61	–	–
Buckwheat	0.51	–	–
White mustard	0.23	–	–
Inter-row (annual species)	RC_i_	Inter-row (perennial species)	RC_i_
Field pea + Cereal rye	0.89	Perennial ryegrass + Tillage radish	0.94
Crimson clover	0.83	Tall fescue + Red fescue + Sheep fescue	0.65
Persian clover	0.82	Crested wheatgrass + Pubescent wheatgrass	0.60
Balansa clover	0.81	Blue grama	0.60
Alsike clover	0.80	Indian ricegrass + Buckwheat	0.35
Berseem clover	0.79	Western wheatgrass	0.08
Hairy vetch + Cereal rye	0.18	Birdsfoot trefoil + Western wheatgrass	0.07
		Canada blue grass	0.06

Top-performing species in each category have higher RC values. RC_i_ = relative closeness to ideal solution (0 to 1, with 1 being ideal).

MCDA, multicriteria decision analysis; AHP, Analytic Hierarchy Process; TOPSIS, Technique for Order Preference by Similarity to Ideal Solution.

^1^ Tillage radish is an annual species that was mixed with a perennial species.

For inter-row covers at CFF Vineyard, field pea + cereal rye was the top annual treatment (RC_i_ = 0.89), followed by annual clovers, which performed more uniformly and at lower levels (RC_i_ = 0.79–0.83) (**Table 8**). The lowest annuals remained hairy vetch + cereal rye (0.18). For perennial inter-row covers at CFF Vineyard, perennial ryegrass + radish outperformed other species (RC_i_ = 0.94). Fescue mixture, crested and pubescent wheatgrass, and blue grama performed similarly with RC_i_ values between 0.60 and 0.65. Indian ricegrass + buckwheat, western wheatgrass with or without birdsfoot trefoil, and Canada blue grass showed the lowest RC_i_ values (0.06–0.08).

### 3.4 Cover crops ranking at Kalala Organic Estate Winery

At KOW Vineyard, spring lentil achieved the highest RC_i_ (0.91) among annuals, making it the top recommended annual in-row cover at both sites (**Table 9**). Close behind were Winfred brassica (RC 0.89) and turnip (RC_i_ = 0.87). Phacelia and field peas were middle-ranked (RC_i_ = 0.61–0.75). Buckwheat and white mustard ranked the lowest (RC_i_ = 0.22–0.50). For perennial in-row options at KOW Vineyard, Ladino white clover clearly outperformed buffalo grass, with RC_i_ = 0.82 *vs*. 0.18.

**Table 9:** Cover crop screening and ranking at Kalala Organic Estate Winery (MCDA AHP–TOPSIS results).

In-row (annual species)	RC_i_	In-row (perennial species)	RC_i_
Spring lentil	0.91	Ladino white clover	0.82
Winfred brassica	0.89	Buffalo grass	0.18
Turnip	0.87	–	–
Phacelia	0.75	–	–
Field pea	0.61	–	–
Buckwheat	0.50	–	–
White mustard	0.22	–	–
Inter-row (annual species)	RC	Inter-row (perennial species)	RC
Berseem clover	0.94	Perennial ryegrass + Tillage radish	0.94
Field pea + Cereal rye	0.91	Tall fescue + Red fescue + Sheep fescue	0.69
Crimson clover	0.88	Crested wheatgrass + Pubescent wheatgrass	0.67
Persian clover	0.85	Blue grama	0.67
Alsike clover	0.84	Indian ricegrass + Buckwheat	0.30
Balansa clover	0.84	Birdsfoot trefoil + Western wheatgrass	0.17
Hairy vetch + Cereal rye	0.11	Western wheatgrass	0.11
		Canada blue grass	0.06

Top-performing species in each category have higher RC values. RC_i_ = relative closeness to ideal solution (0 to 1, with 1 being ideal).

MCDA, multicriteria decision analysis; AHP, Analytic Hierarchy Process; TOPSIS, Technique for Order Preference by Similarity to Ideal Solution.

^1^ Tillage radish is an annual species that was mixed with a perennial species.

In the inter-row category for KOW Vineyard, the highest-ranked treatment was berseem clover with an outstanding RC_i_ of 0.94 (**Table 9**). The second-best species was field pea + cereal rye (RC_i_ = 0.91), followed by the rest of the annual clovers, which performed similarly (RC_i_ = 0.84–0.88). Hairy vetch + rye scored the lowest (RC_i_ = 0.11) at KOW Vineyard. For inter-row perennial species, perennial ryegrass + tillage radish performed the best (RC_i_ = 0.94) at KOW Vineyard. Fescue mixture, crested and pubescent wheatgrass, and blue grama performed similarly with RC_i_ values between 0.67 and 0.69, while Indian ricegrass + buckwheat, western wheatgrass with or without birdsfoot trefoil, and Canada blue grass showed the lowest RC_i_ values (0.06–0.30).

### 3.5 Sensitivity analysis—influence of invasiveness criterion

Given that the risk of being invasive was the most influential criterion in our model (weight = 0.432–0.596), the AHP–TOPSIS model was re-run with the risk of being invasive criterion removed (weight set to 0 and the remaining weights re-normalized) (**Supplementary Data Table S6**). The results showed that with this change, the relative importance of other criteria shifted. For in-row annual covers, “Interference with fruiting zone” became dominant (weight = 0.67 for annuals), while “ground coverage” and “biomass” carried modest weights (0.06–0.25 and 0.10–0.53, respectively).

At CFF Vineyard, the rankings did not notably change for the top performer when invasiveness risk was excluded. Among in-row annuals, spring lentil led (RC_i_ = 0.94), ahead of turnip (0.90) and Winfred brassica (0.88) (**Supplementary Data Table S7**). For in-row perennials, Ladino white clover again topped the list (0.98), while buffalo grass remained low (0.02). However, the ranking shift in inter-row cover crop species was more pronounced. In the inter-row annual mixes, hairy vetch + cereal rye became the top-ranked option (RC_i_ = 0.92), with field pea + rye second (0.77). Among inter-row perennials, perennial ryegrass + tillage radish remained the highest (0.97), followed by Indian ricegrass + buckwheat (0.67).

At KOW Vineyard, for instance, white clover, which had a minor issue with invasiveness in some blocks but overall was not aggressive, saw its RC_i_ jump to 0.98 (from 0.82) and became the top in-row perennial by an even larger margin (**Supplementary Data Table S8**). Conversely, species like hairy vetch improved in score but remained low-ranked due to other issues. The biggest differences were observed in species that previously had moderate scores, but a sizable invasiveness penalty: for example, white mustard’s RC_i_ went from 0.22 to 0.48, or Indian ricegrass + buckwheat RC_i_ rose from 0.30 to 0.64 at KOW Vineyard when invasiveness was omitted, reflecting that invasiveness was a major flaw in these species.

## 4 Discussion

The present study reinforces several established vineyard cover-cropping principles while providing new, site-specific insights. Legume-based cover crops often perform strongly, especially as in-row vegetation, because they can moderate vigor while improving soil function (Vanden Heuvel et al., 2021; Sharifi et al., 2024). The results indicated that species such as clovers and lentils contribute biologically fixed N and organic matter, while their low-growing habit makes them suitable for under-trellis management (Patrick et al., 2004; Ovalle et al., 2010). These findings are consistent with long-term South African trials showing that N-fixing legumes can enhance N status and sustain yield depending on soil context (Fourie, 2010, 2011). Our TOPSIS analysis identified Ladino white clover as the top perennial in-row cover crop (RC_i_ ≈ 0.8), aligning with the established use of clover cultivars (including Ladino types) in New Zealand and European vineyards, particularly in organic and low-herbicide systems (Merfield, 2019; Gough et al., 2025). In addition to the perennial clover outcome, spring lentil emerged as a viable annual under-vine candidate under the tested conditions. Although lentils are rarely used in vineyards, our field results identify spring lentil as a high-performing annual under-vine cover, aligning with a recent greenhouse screening by Sharifi et al. (2024). Compared with peas or viny vetches, lentil’s short, semi-erect habit reduces trellis climbing and fruit-zone interference while still providing N fixation (Sattell, 1998; Hofer et al., 2009). Lentil can self-reseed via pod drop, offering limited natural persistence if tolerated (USDA-NRCS, 2015; USA Dry Pea & Lentil Council, 2018). Notably, small-stature lentil cultivars released in Saskatchewan (~20–40 cm) appear particularly well-suited for under-trellis conditions in semi-arid vineyards (USask CDC, 2023). Related small-seeded legumes such as chickling vetch (*Lathyrus sativus*) and fenugreek (*Trigonella foenum-graecum*) share similar traits and merit future testing in this context (USDA-NRCS, 2016). In contrast to the low-stature legumes, species with climbing (e.g., hairy vetch and field pea) or excessive height (e.g., buckwheat and mustards) performed poorly under trellis and should be avoided or terminated early before approaching the cluster zone. Dense and tall vegetation beneath the vines may be undesirable for disease management (e.g., powdery mildew and bunch-rot) and fruit exposure (Jordan et al., 2016).

The pea–cereal rye mixture demonstrated superior inter-row performance, attaining an RC_i_ of 0.95 at KOW Vineyard and ranking among the highest treatments across sites. This outcome is consistent with complementary functional traits such as rapid, weed-suppressive canopy and rooting by rye coupled with symbiotic N inputs and additional biomass from pea, yielding greater multifunctionality than monocultures (Finney et al., 2017; Akemo et al., 2000; Florence and McGuire, 2020). Cereal–legume mixes commonly balance carbon- and N-driven processes, with cereals adding carbon-rich biomass and legumes supplying biologically fixed N, thereby supporting soil structure and nutrient cycling (Chapagain et al., 2020; Van Eerd et al., 2023). Guerra and Steenwerth (2012) likewise highlighted cereal–legume mixtures in vineyards as beneficial for soil fertility and microbial activity without compromising vine nutrition, noting context-dependent responses. This two-species mixture appears to be a robust inter-row option across diverse Okanagan microclimates, in line with regional vineyard studies evaluating cover crops in the Northwest semi-arid conditions (Olmstead et al., 2001; Sharifi et al., 2024; Sharifi and Zolfaghari, 2025). Under inter-row conditions, perennial ryegrass with nurse crops (e.g., tillage radish or oats) delivered rapid ground cover and early weed suppression, while crimson clover, despite literature support, proved too drought-sensitive to be a reliable inter-row candidate in semi-arid, irrigated vineyards. Common vetch and faba bean (*Vicia faba* L.) may be viable substitutes only under strict management, echoing South African trials (Fourie, 2010, 2011; Weil et al., 2009). Cover crops increase water demand and alter the vineyard water balance, especially in arid or Mediterranean climates. If mismanaged, they can promote pests and diseases and off-phase vine vigor that reduce fruit quality; therefore, irrigation and termination should be tailored to site conditions (Celette et al., 2008).

Based on the AHP criteria, sensitivity to insect damage ranked third in weight after invasiveness and interference with the fruiting zone. Cover crops can serve as habitat and resources for pollinators, parasitoids, and predators (Rusch et al., 2016). In Québec, Canada, emerging work indicates that perennial in-row covers can contribute to biological control and beneficial arthropod communities in cool-climate vineyards (CETAB, 2023; Denis, 2023). In Italy, lower European grapevine moth (*Lobesia botrana*) infestation has been reported in cover-cropped inter-rows compared with tilled vineyards (Serra et al., 2006). When arthropods are sampled from vine canopies, fully vegetated inter-rows tend to host higher abundances, and wild bee activity increases with flower cover (Blaise et al., 2022). Although purple top turnip and Winfred brassica showed some insect infestation, the insects were not significant grapevine pests; therefore, these species ranked highly in the TOPSIS analysis.

Biomass production received a low weight in the AHP criteria, reflecting a preference for moderate-growth cover crops that limit invasiveness, resource competition, and vine interference, consistent with sustainability principles that balance ecosystem services with manageable competition (Christ and Burritt, 2013; Abad et al., 2021a, b). White mustard, despite high biomass potential, was thus less preferred due to volunteer self-seeding risk, whereas moderate-biomass covers like white clover are often favored for manageability in in-row settings. The high weighting on invasiveness reflects concerns that hard-seeded or climbing species can form persistent seedbanks or reach the fruiting zone if termination is delayed, increasing long-term control costs and canopy-management risks (McKenzie-Gopsill et al., 2025). Invasive-prone covers may also intensify competition for vine water and N, potentially reducing vigor and yield in dry environments (Celette et al., 2008; Celette and Gary, 2013). Recent syntheses show that cover crops frequently improve soil condition and ecosystem functions, but effects on vine vigor and yield are context-dependent, necessitating site-specific termination strategies that prevent seed set and minimize competition (García et al., 2024; Liebhard et al., 2024). These concerns are particularly significant in organic vineyards lacking herbicidal options, reinforcing the need to prioritize species with low invasiveness and to rely on robust mechanical termination (Ramírez-García et al., 2015; García et al., 2024).

This study advances whole-floor screening (inter-row and in-row) via an MCDA–AHP framework, generating site-specific field data that identified promising species. Key limitations include the single-season scope (insufficient for perennial trajectories), the absence of direct vine response metrics, and the small-plot scale. AHP weights are context-dependent and subjective (Ishizaka and Labib, 2011; Velasquez and Hester, 2013), and the linear-additive model assumes criterion independence and is prone to rank reversal (Triantaphyllou, 2001; Ishizaka and Labib, 2011). Even so, the concordant rankings across sites indicate practical robustness and support multi-site/on-vineyard validation to strengthen generalizability.

## 5 Conclusion

This study identified key criteria and their corresponding weights for selecting cover-crop species and demonstrated a successful hybrid MCDA (AHP–TOPSIS) application to rank suitable options for irrigated vineyards in the Okanagan Valley, Canada. Top-ranked species included spring lentil, Winfred brassica, and purple top turnip for in-row annual covers; Ladino white clover for perennial in-row cover; and a field pea–cereal rye mixture for inter-row covers. Perennial ryegrass combined with tillage radish showed promise for long-term inter-row cover. The analysis confirmed the importance of selecting cover crops that successfully establish and minimize invasiveness and vine interference. The hybrid MCDA model provides a replicable and adaptable framework for agricultural decision-making, valuable for regions transitioning toward sustainable vine management practices. Future studies should include multi-year trials to quantify long-term soil and vine responses, incorporate economic criteria (seed, establishment, and management costs), validate the MCDA framework across diverse climates and vineyard systems, integrate explicit biodiversity and ecosystem service metrics, and develop user-friendly, web-based decision-support tools built on expanded datasets.

## Data Availability

The original contributions presented in the study are included in the article/**Supplementary Material**. Further inquiries can be directed to the corresponding author.
