# Supplementary material for: Defining screening criteria and ranking in-row and inter-row cover crops for irrigated vineyards using a hybrid AHP–TOPSIS model
Source: Front Plant Sci. 2026 Jan 26;16:1695610. doi: 10.3389/fpls.2025.1695610 (PMC12884541; doi:10.3389/fpls.2025.1695610)
Supplement: Supplementary file 1 [file Table1.docx]

**Supplementary Data**

**Defining screening criteria and ranking in-row and inter-row cover crops for irrigated vineyards using a hybrid AHP–TOPSIS model**

Table S1 Summary of descriptions and management histories for the selected study vineyards. Both vineyards are located in the Okanagan Valley, British Columbia, Canada.

| **Site** | **Latitude and Longitude (^o^N^, o^W)** | **Growing season rainfall^†^ (mm)** | **Mean growing season air temperature**^1^ **(^o^C)** | **Fertilizer Rates applied (kg ha^-1)^** | **Starter N applied (kg N ha^-1)^** | **Grape variety** | **Soil type** | **Management practices** |
| --- | --- | --- | --- | --- | --- | --- | --- | --- |
| Covert Family Farm Estate | 49°14'39.8"  119°32'42.7" | 134 | 10.7 | None applied | None applied | 13-yr old Merlot | Loamy sand | Drip irrigation, undercanopy sprinkler |
| Kalala Organic Estate Winery | 49°50'31.2"  119°38'42 | 143 | 18.6 |  |  | 10-yr old Zweigelt | Sandy loam | Drip irrigation, undercanopy sprinkler |

^1^ From May to October: <https://climate.weather.gc.ca/historical_data/search_historic_data_e.html>

Table S2 Mean and range (in parentheses: min, max) of soil properties sampled at a depth of 0–15 cm at each study site. Samples were collected before seeding in May 2019 (n = 4).

| **Site** | **Electerdial Conductivity (μS/cm)** | **pH** | **%C** | **%N** | **C/N** | **% Clay** | **% Silt** | **% Sand** | **Classifi-cation** |
| --- | --- | --- | --- | --- | --- | --- | --- | --- | --- |
| Covert Family Farm Estate | 148  (115, 167) | 6.34  (6.30, 6.40) | 0.813  (0.510, 1.356) | 0.0716  (0.0446, 0.1199) | 11.500  (10.413, 13.220) | 6.00  (4.67, 6.87) | 9.72  (6.30, 12.39) | 84.27  (80.73, 89.03) | Loamy sand |
| Kalala Organic Estate Winery | 262  (70, 91) | 6.31  (6.03, 6.63) | 1.492  (0.958, 2.075) | 0.1258  (0.0865, 0.1678) | 11.864  (11.073, 12.617) | 14.00  (11.72, 16.12) | 32.64  (27.39, 37.33) | 53.36  (46.55, 60.89) | Sandy loam |

Table S3 The effect of the criteria (Level 3) in TOPSIS calculations.

| **No** | **Criteria (Level3)** | **Effect** |
| --- | --- | --- |
| 1 | Total dry biomass | Beneficial |
| 2 | Ground coverage | Beneficial |
| 3 | Interfere with grape fruiting zone | Non-beneficial |
| 4 | Risk of being invasive | Non-beneficial |
| 5 | Harboring pests | Non-beneficial |
| 6 | Drought tolerance | Beneficial |
| 7 | Winter Hardiness (HRS) | Beneficial |
| 8 | Resistance to traffic after germination | Beneficial |

Table S4 Separation measures from ideal solution for in-row cover crop species at Covert Family Farm Estate (CFFE) and Kalala Organic Estate Winery (KOEW).

| **Annual alternatives**  **(Level 3)** | **d_i_^+^** | | **d_i_^-^** | **d_i_^+^** | **d_i_^-^** |
| --- | --- | --- | --- | --- | --- |
|  | CFFE | | | KOEW | |
| Spring Lentil | 0.01 | | 0.16 | 0.02 | 0.16 |
| Turnip | 0.04 | | 0.16 | 0.04 | 0.16 |
| Winfred Brassica | 0.04 | | 0.16 | 0.04 | 0.16 |
| Phacelia | 0.06 | | 0.14 | 0.06 | 0.14 |
| Field Pea | 0.11 | | 0.13 | 0.11 | 0.13 |
| Buckwheat | 0.16 | | 0.04 | 0.16 | 0.04 |
| White Mustard | 0.16 | | 0.04 | 0.16 | 0.04 |
| **Prennial alternatives**  **(Level 3)** |  |  | |  |  |
| Crescendo Ladino Clover | 0.01 | 0.04 | | 0.01 | 0.03 |
| Buffalo Grass | 0.04 | 0.01 | | 0.03 | 0.01 |

Note: d_i_^+^ is the separation measure from the PIS and d_i_^-^ is the separation measure from the NIS. PIS is the positive ideal solution and NIS is negative ideal solution.

Table S5 Separation measures from ideal solution for inter-row cover crop species at Covert Family Farm Estate (CFFE) and Kalala Organic Estate Winery (KOEW).

| **Annual alternatives**  **(Level 3)** | **d_i_^+^** | **d_i_^-^** | **d_i_^+^** | **d_i_^-^** |
| --- | --- | --- | --- | --- |
|  | CFFE | | KOEW | |
| Field Pea+Cereal Rye | 0.02 | 0.20 | 0.01 | 0.19 |
| Crimson Clover | 0.06 | 0.19 | 0.02 | 0.19 |
| Balansa Clover | 0.07 | 0.19 | 0.04 | 0.19 |
| Persian Clover | 0.07 | 0.19 | 0.05 | 0.19 |
| Alsike Clover | 0.07 | 0.19 | 0.05 | 0.19 |
| Berseem Clover | 0.08 | 0.19 | 0.06 | 0.19 |
| Hairy Vetch+Cereal Rye | 0.19 | 0.07 | 0.19 | 0.04 |
| **Prennial alternatives**  **(Level 3)** |  |  |  |  |
| Perennial Ryegrass+Tillage Radish | 0.04 | 0.20 | 0.04 | 0.19 |
| Tall Fescue+Red Fescue+Sheep Fescue | 0.07 | 0.18 | 0.06 | 0.18 |
| Crested Wheatgrass + Pubescent Wheatgrass | 0.09 | 0.18 | 0.06 | 0.18 |
| Blue grama | 0.09 | 0.18 | 0.06 | 0.18 |
| Birdsfoot Trifoile +Western Wheatgrass | 0.09 | 0.18 | 0.07 | 0.18 |
| Western Wheatgrass | 0.09 | 0.18 | 0.07 | 0.18 |
| Canada Blue Grass | 0.09 | 0.18 | 0.08 | 0.18 |
| Indian Ricegrass+ Buckwehat | 0.18 | 0.08 | 0.18 | 0.07 |

Note: d_i_^+^ is the separation measure from the PIS and d_i_^-^ is the separation measure from the NIS. PIS is the positive ideal solution and NIS is negative ideal solution.

Table S6 AHP-derived importance weights for cover crop selection criteria when the ‘Risk of being invasive’ feature is excluded. Separate analyses were conducted for in-row and inter-row contexts; criteria not applicable in a given context are marked as N/A.

| **Criteria** | **In-Row** | | **Inter-Row** | |
| --- | --- | --- | --- | --- |
|  | Annual | Perennial | Annual | Perennial |
| Cover crop dry matter (total biomass) | 0.062 | 0.255 | 0.210 | 0.200 |
| Cover crop ground coverage | 0.098 | 0.533 | 0.491 | 0.519 |
| Interfere with fruiting zone | 0.674 | 0.045 | N/A | N/A |
| Sensitivity to insect damage | 0.129 | 0.123 | 0.115 | 0.123 |
| Drought tolerance | 0.026 | 0.033 | 0.013 | 0.033 |
| Plant Winter Hardiness (HRS) | 0.011 | 0.011 | 0.039 | 0.011 |
| Resistance to traffic | N/A | N/A | 0.133 | 0.114 |

Table S7 Cover crop screening and ranking at Covert Family Farm Estate (MCDA AHP–TOPSIS results) when the ‘Risk of being invasive’ feature is excluded. Top-performing species in each category (highest RC) are listed first. RC = relative closeness to the ideal solution (0 to 1, with 1 being ideal).

| **In-row Annual** | **RC ^1^** | **In-row Perennial** | **RC** |
| --- | --- | --- | --- |
| Spring Lentil | 0.94 | Crescendo Ladino Clover | 0.98 |
| Turnip | 0.90 | Buffalo Grass | 0.02 |
| Winfred Brassica | 0.88 |  |  |
| Buckwheat | 0.51 |  |  |
| Phacelia | 0.50 |  |  |
| White Mustard | 0.49 |  |  |
| Field Pea | 0.07 |  |  |
| **Inter-row Annual** | **RC** | **Inter-row Perennial** | **RC** |
| Hairy Vetch+Cereal Rye | 0.92 | Perennial Ryegrass+Tillage Radish | 0.97 |
| Field Pea+Cereal Rye | 0.77 | Indian Ricegrass+ Buckwheat | 0.67 |
| Crimson Clover | 0.30 | Tall Fescue+Red Fescue+Sheep Fescue | 0.24 |
| Persian Clover | 0.20 | Blue grama | 0.11 |
| Balansa Clover | 0.09 | Crested Wheatgrass + Pubescent Wheatgrass | 0.10 |
| Berseem Clover | 0.08 | Birdsfoot Trifoile +Western Wheatgrass | 0.09 |
| Alsike Clover | 0.03 | Western Wheatgrass | 0.07 |
|  |  | Canada Blue Grass | 0.07 |

Table S8 Cover crop screening and ranking at Kalala Organic Estate Winery (MCDA AHP–TOPSIS results) when the ‘Risk of being invasive’ feature is excluded. Top-performing species in each category (highest RC) are listed first. RC = relative closeness to the ideal solution (0 to 1, with 1 being ideal).

| **In-row Annual** | **RC ^1^** | **In-row Perennial** | **RC** |
| --- | --- | --- | --- |
| Winfred Brassica | 0.90 | Crescendo Ladino Clover | 0.96 |
| Spring Lentil | 0.87 | Buffalo Grass | 0.04 |
| Turnip | 0.86 |  |  |
| Buckwheat | 0.49 |  |  |
| Phacelia | 0.49 |  |  |
| White Mustard | 0.48 |  |  |
| Field Pea | 0.07 |  |  |
| **Inter-row Annual** | **RC** | **Inter-row Perennial** | **RC** |
| Berseem Clover | 0.78 | Perennial Ryegrass+Tillage Radish | 0.96 |
| Field Pea+Cereal Rye | 0.77 | Indian Ricegrass+ Buckwehat | 0.64 |
| Hairy Vetch+Cereal Rye | 0.57 | Birdsfoot Trifoile +Western Wheatgrass | 0.34 |
| Crimson Clover | 0.52 | Blue grama | 0.30 |
| Persian Clover | 0.13 | Tall Fescue+Red Fescue+Sheep Fescue | 0.29 |
| Alsike Clover | 0.06 | Crested Wheatgrass + Pubescent Wheatgrass | 0.23 |
| Balansa Clover | 0.04 | Western Wheatgrass | 0.18 |
|  |  | Canada Blue Grass | 0.08 |
